# Hydrogen Peroxide Signaling in the Maintenance of Plant Root Apical Meristem Activity

**DOI:** 10.3390/antiox13050554

**Published:** 2024-04-30

**Authors:** Hui Liu, Yangwei Mu, Yuxin Xuan, Xiaolin Wu, Wei Wang, Hui Zhang

**Affiliations:** National Key Laboratory of Wheat and Maize Crop Science, College of Life Sciences, Henan Agricultural University, Zhengzhou 450046, China; liuhuisw@henau.edu.cn (H.L.); muyw@stu.henau.edu.cn (Y.M.); xyxin@stu.henau.edu.cn (Y.X.); wuxiaolin@henau.edu.cn (X.W.)

**Keywords:** hydrogen peroxide, root apical meristem, stem cell niche, quiescent center, auxin

## Abstract

Hydrogen peroxide (H_2_O_2_) is a prevalent reactive oxygen species (ROS) found in cells and takes a central role in plant development and stress adaptation. The root apical meristem (RAM) has evolved strong plasticity to adapt to complex and changing environmental conditions. Recent advances have made great progress in explaining the mechanism of key factors, such as auxin, WUSCHEL-RELATED HOMEOBOX 5 (WOX5), PLETHORA (PLT), SHORTROOT (SHR), and SCARECROW (SCR), in the regulation of RAM activity maintenance. H_2_O_2_ functions as an emerging signaling molecule to control the quiescent center (QC) specification and stem cell niche (SCN) activity. Auxin is a key signal for the regulation of RAM maintenance, which largely depends on the formation of auxin regional gradients. H_2_O_2_ regulates the auxin gradients by the modulation of intercellular transport. H_2_O_2_ also modulates the expression of *WOX5*, *PLTs*, *SHR*, and *SCR* to maintain RAM activity. The present review is dedicated to summarizing the key factors in the regulation of RAM activity and discussing the signaling transduction of H_2_O_2_ in the maintenance of RAM activity. H_2_O_2_ is a significant signal for plant development and environmental adaptation.

## 1. Introduction

Roots are important structures for plant growth, development, and environmental adaptation. It is well known that roots have many functions, such as the uptake and transport of water and nutrients, synthesis and secretion of compounds, storage of substances, and anchorage of the plant. These functions help plants to better adapt to the environment. The external morphology of the root tip of Arabidopsis reveals the presence of three distinct zones, namely the meristematic zone, elongation zone, and differentiation zone. There is a transition zone between the meristematic zone and the elongation zone, where cells leave the meristem and enter the elongation zone. The region where the cortical cells begin to expand is the transition zone. The transition zone is a key point in the study of cell division and cell differentiation [1]. In the differentiation zone, elongated cells differentiate to maturity.

**Figure 1:**
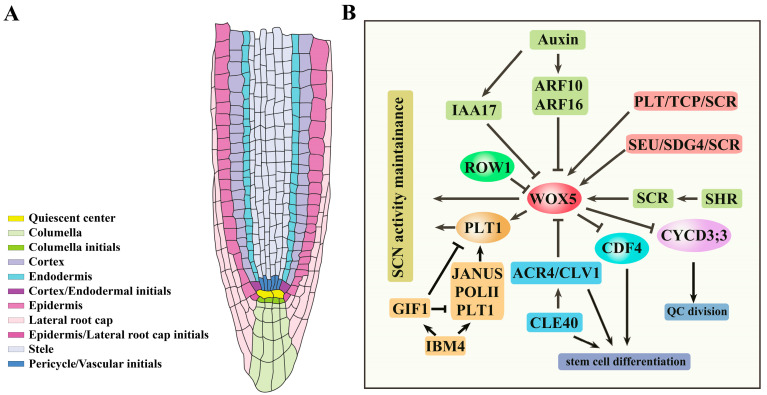
Key signaling in the regulation of the root apical meristem. (**A**). The structure of the root apical meristem (RAM). The quiescent center (QC) and its surrounding stem cells together form a stem cell niche (SCN). Different stem cells (also called initials) produce a variety of cell types in roots. (**B**). The key signals for controlling SCN maintenance. WOX5 is a critical factor for the regulation of SCN maintenance. Auxin represses *WOX5* expression by ARF10, ARF16, and IAA17. ROW1 and the module CLE40/ACR4/CLV1 can also inhibit *WOX5* expression. The modules SHR/SCR, PLT/TCP/SCR, and SEU/SDG4/SCR promote *WOX5* expression. In addition, WOX5 promotes *PLT1* expression to control SCN activity. GIF1 and JANUS are antagonistic to regulate *PLT1* expression (**B**).

The root apical meristem (RAM), situated within the meristematic zone, plays a crucial role in controlling root growth and development. The quiescent center (QC) and its surrounding stem cells together form a stem cell niche (SCN), providing continuous cell sources for root growth and development. Different stem cells (also called initials), including pericycle/vascular initials, cortex/endodermal initials, epidermis/lateral root cap initials, and columella initials (also called columella stem cells, or CSCs), produce a variety of cell types in roots (Figure 1A) [1]. These distinct stem cell populations are responsible for generating various cell types through processes of division and differentiation. Specifically, pericycle/vascular initials contribute to the formation of stele cells and pericycle cells, cortex/endodermal initials give rise to the endodermis and cortex, epidermis/lateral root cap initials are responsible for the development of the epidermis and lateral root cap, and columella initials contribute to the formation of columella cells.

The phytohormone auxin has been shown to be a central player in multiple biological processes, such as root development, embryo development, and leaf development [2,3,4,5]. During root development, auxin plays an essential role in controlling the organization and function of RAM [4,6]. *WUSCHEL-RELATED HOMEOBOX 5* (*WOX5*) is a HOMEOBOX family gene that is specifically expressed in the QC and plays a crucial role in determining the fate of stem cells. It is generally believed that the establishment and maintenance of the SCN microenvironment are strictly controlled by two core transcription factor pathways: the longitudinal PLETHORA (PLT) pathway and the radial SHORTROOT (SHR)/SCARECROW (SCR) pathway [7].

Higher plants continuously adjust their developmental processes to adapt to complex and changing environmental conditions. The evolution of roots allows vascular plants to adapt to land environments, and some common molecular modules in RAM are employed during root origins in lycophytes and euphyllophytes [8]. Compared to animals, plant roots exhibit strong adaptation in the processes of growth and development, which mainly depend on the activity of SCN in the RAM. RAM organization has evolved to become highly plastic and dynamic in response to environmental triggers, such as water and nutrient availability [9,10]. Under stress conditions, the RAM is rapidly reorganized, and the root growth is enhanced, thus providing effective strategies to cope with the stresses [9,10,11]. This environment-responsive developmental plasticity is linked to reactive oxygen species (ROS) and nitric oxide (NO), which act as signals specifically to regulate RAM function, connecting with hormones [10,12,13]. ROS and NO are considered key players in RAM maintenance and environmental stress responses [14,15,16,17].

ROS, including hydrogen peroxide (H_2_O_2_), superoxide (O_2_^•−^), singlet oxygen (^1^O_2_), and hydroxyl radical (·OH), are considered to be toxic byproducts of metabolism that could harm cells [18]. A high level of ROS results in serious damage to cell structures and cell death. However, many studies have demonstrated that ROS play a significant role in various biological processes when at appropriate concentrations [19]. Among these ROS, H_2_O_2_ takes a central role in plant development and stress response [20]. H_2_O_2_ serves as a membrane-permeable and diffusible molecule that is less reactive and has a longer lifespan compared to the highly reactive ·OH. This characteristic makes H_2_O_2_ well-suited for functioning as a signaling molecule [20]. The production of H_2_O_2_ can occur through intracellular and extracellular pathways. Plant cells employ an antioxidant system to regulate H_2_O_2_ levels, thereby ensuring the maintenance of a suitable redox environment conducive to cellular growth [21].

ROS are key signals in the regulation of elongation and differentiation in plant roots. Studies on Arabidopsis have shown that O_2_^•−^ is mainly distributed in the extracellular vesicles of the elongation zone, while H_2_O_2_ accumulates in the cell walls of differentiated tissues and root hairs. The decrease in O_2_^•−^ concentration can reduce root elongation and root hair formation, but removing H_2_O_2_ can promote root elongation. The balance between O_2_^•−^ and H_2_O_2_ regulates the transition of root systems between proliferation and differentiation [22]. Recent advances indicate that H_2_O_2_ functions as a signaling molecule to control RAM size and SCN maintenance by regulating the factors of auxin, PLTs, SCR, and SHR [11,23,24,25]. In this review, we focus on summarizing the key factors in the regulation of RAM activity and discuss the signaling transduction of H_2_O_2_ in the maintenance of RAM activity.

## 2. Key Factors in the Maintenance of RAM Activity

**Figure 2:**
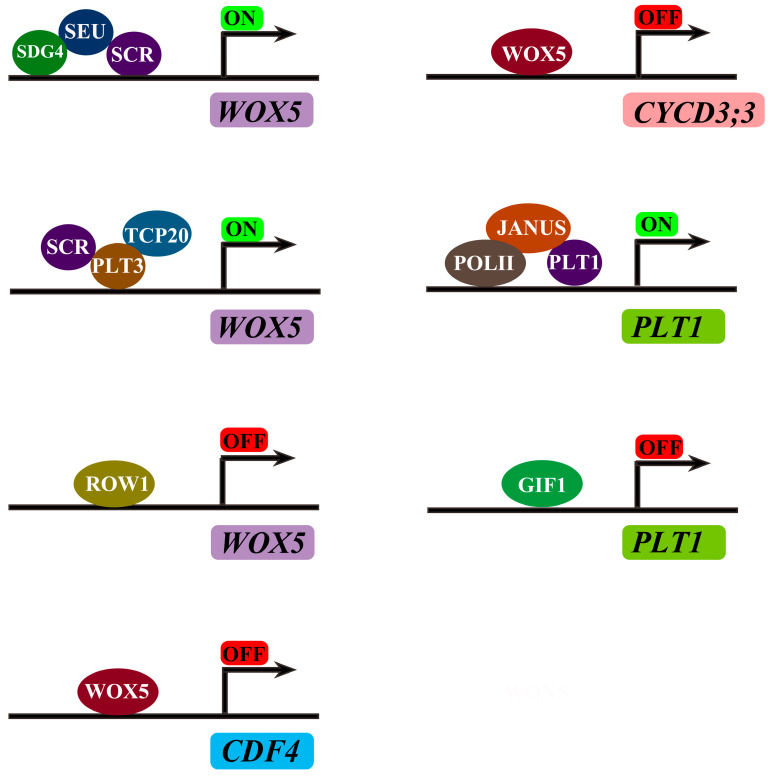
Direct regulation of the key genes involved in root apical meristem. The modules SEU/SCR/SDG4 and PLT3/SCR/TCP20 directly bind to the promoter of *WOX5* to activate its expression. ROW1 directly represses *WOX5* expression. WOX5 can inhibit *CDF4* and *CYCD3;3* expression by binding to their promoters. The module PLT1/JANUS/POLII directly promotes *PLT1* expression. GIF1 functions to repress the expression of *PLT1*.

The homeodomain transcription factor gene *WOX5* is specially expressed in the QC to control the CSC identity (Table 1). Loss of function of *WOX5* causes differentiation in CSCs, while increased *WOX5* expression inhibits the differentiation of CSCs and causes CSC over-proliferation [26]. WOX5 inhibits QC division by repressing the expression of the D-type cyclin genes *CYCD3;3* and *CYCD1;1*. In particular, the WOX5 protein can directly interact with the promoter of *CYCD3;3* [27]. It is necessary that WOX5 suppresses CSC differentiation and maintains CSC identity. Another study indicated that the WOX5 protein moves from the QC into CSCs, where it directly represses the transcription factor gene *CYCLING DOF FACTOR 4* (*CDF4*). WOX5 recruits TPL/TPR co-repressors and the histone deacetylase HDA19 to the *CDF4* gene promoter and regulates *CDF4* expression. CDF4 antagonizes WOX5 activity and promotes columella cell differentiation. WOX5 functions as a mobile signal from the QC to the CSC to maintain CSC identity by the modulation of *CDF4* (Figure 1B and Figure 2) [28]. Differentiated columella cells and CSCs provide the secretion peptide CLAVATA3/ESR-RELATED40 (CLE40) as a feedback signal to control *WOX5* expression by the receptor kinases ARABIDOPSIS CRINKLY4 (ACR4) and CLAVATA1 (CLV1) [29,30]. CLV1 can sense the signal of CLE40 by forming dimers or tetramers with ACR4 and negatively regulate the expression of *WOX5* to maintain the activity of CSCs (Figure 1B and Table 1) [30,31]. The PHD domain-containing protein REPRESSOR OF WUSCHEL1 (ROW1) directly represses *WOX5* expression by histone modification [32]. These findings indicate that multiple players are involved in the regulation of the core factor WOX5 to maintain RAM activity (Figure 1B and Figure 2).

Auxin, along with many other hormones, is a key signal in the regulation of RAM activity [4]. The function of auxin depends on its biosynthesis, transport, and signaling transduction [33]. Auxin levels are critical for the maintenance of QC and SCN activities and RAM size. The auxin maximum is located in the SCN region, which is determined by the auxin-responsive reporters [34,35]. The formation of auxin regional gradients depends on the local biosynthesis at the root tips and intercellular transport [4,36,37]. The PIN proteins are responsible for the polar transport of auxin toward the SCN [4,38]. Auxin gradients play a central role in the regulation of SCN identity. *WOX5* is repressed by auxin response factors ARF10 and ARF16 [39]. The QC-localized auxin maximum requires an auxin response repressor indole-3-acetic acid 17 (IAA17), which is responsible for restricting the expression of *WOX5* to maintain root stem cell identity (Figure 1B) [40].

**Table 1:** Expression patterns of the genes involved in RAM maintenance.

Gene	Expression Patterns	References
*WOX5*	*WOX5* is expressed in the quiescent center (QC). WOX5 protein can move into the columella stem cells to repress *CDF4* expression.	[28,39]
*CDF4*	*CDF4* is expressed in the upper differentiated columella cells and the columella stem cells.	[28]
*CLE40*	*CLE40* is expressed in the stele and the differentiated columella cells.	[30]
*ACR4*	*ACR4* is expressed mainly in the three cell layers (D1, D2, and D3) below the quiescent center, adjacent lateral root cap, and epidermis initial, but only occasionally and at a low level in the quiescent center.	[30]
*CLV1*	*CLV1* is expressed in the two cell layers (D1 and D2) immediately distal to the QC, the epidermis/lateral root cap initials, and the lateral root cap.	[31]
*PLT1*	*PLT1* is expressed in the QC, surrounding stem cells, and uppermost layer of differentiated columella cells.	[41]
*PLT2*	*PLT2* displays the same expression pattern as *PLT1*.	[41]
*PLT3*	*PLT3* is expressed in the QC and vascular initial cells.	[42,43]
*SCR*	*SCR* is primarily synthesized in the endodermis, QC, and cortex endodermal initials.	[44]
*SHR*	*SHR* is expressed in the stele cells internal to the endodermis and cortex. The SHR protein can move from the stele to a single layer of adjacent cells.	[45]
*CYCD3;3*	*CYCD3;3* is expressed in the columella layers, the lateral root cap, the epidermal stem cells, and the stele.	[27]

The AP2-domain transcription factors PLTs are key factors that control RAM maintenance. Four PLTs, namely, *PLT1*, *PLT2*, *PLT3,* and *PLT4* (also known as BBM), are essential for QC specification and stem cell activity as the multiple *plt* mutants cannot form RAM [41,42,46]. High levels of PLT activity promote stem cell identity and maintenance, while low levels of PLT activity promote mitotic activity in stem cell daughters [42]. The PLT proteins form a graded distribution with a maximum near the root tip, which is critical for QC specification and SCN identity (Table 1) [46]. Many factors have been identified to control the expression of *PLTs* in the root meristem [47,48]. The transcriptional coactivator GRF-INTERACTING FACTOR1 (GIF1), also known as ANGUSTIFOLIA3 (AN3), negatively regulates the expression of *PLT1* by directly binding to the promoter of *PLT1* [48]. JANUS, interacting with PLT1, positively regulates *PLT1* expression in the root meristem by recruiting RNA polymerase II (Pol II) on *PLT1*. JANUS-dependent recruitment of Pol II is inhibited through competitive binding of JANUS by GIF1. GIF1 and JANUS, the antagonistic regulators of PLT1, both depend on IMPORTIN β4 (IMB4) for their nuclear accumulation. The strong signals of GFP-GIF1 and JANUSg-GFP have been detected in the nuclei of wild type but not in the *imb4-1* mutant. IMB4 directly interacts with GIF1 and regulates the nucleocytoplasmic transport of GIF1, possibly by an unknown protein modification [49]. IMB4 positively regulates the nuclear accumulation of JANUS by preventing 26S proteasome-mediated degradation (Figure 1B and Figure 2) [47]. The salt overly sensitive (SOS) pathway component SOS2 interacts and phosphorylates PLT1 and PLT2 through their conserved C-terminal motifs to stabilize PLT1 and PLT2 to regulate root apical meristem maintenance for adaptation to salt stress [50]. Pre-mRNA (messenger RNA) splicing participates in the regulation of RAM activity. *RDM16* encodes Prp3, a component of U4/U6 snRNP involved in pre-mRNA splicing in Arabidopsis. The mutation of *RDM16* results in the abnormal pre-mRNA splicing of *PLT1* and *PLT2*, leading to the disordering of the root stem cell niche and a short-root phenotype [51]. WOX5 and PLT proteins overlap in the SCN, and PLT1, PLT2, PLT3, and PLT4 contain PrD domains. Using fluorescence lifetime imaging microscopy (FLIM), the researchers confirmed that WOX5 interacts with PLTs, including PLT1, PLT2, PLT3, and PLT4. Another study indicated that PrD domains of PLT3 are required for the interaction of WOX5 and are necessary for the maintenance of the SCN. WOX5 forms a complex with PLTs to control stem cell identity [52].

Precise control of cell division is essential for root development. The transcription factors SHR and SCR are required for the formative division in the RAM of Arabidopsis roots. Both SHR and SCR belong to the GRAS family of transcription factors. SHR is expressed in the stele, while SCR is primarily synthesized in the endodermis, QC, and cortex endodermal initials. The movement of the SHR protein to the single layer of adjacent cells regulates the specification of endodermal cell identity and enhances *SCR* expression [44,45,53]. *SCR* expression in QC is crucial for the maintenance of the root SCN. The SCR protein directly interacts with the SHR protein, confining SHR to the nucleus (Table 1) [44,53]. Recently, a cell-resolution map of the maize root was generated, revealing an alternative configuration of the tissue formative SHR adjacent to an expanded cortex. The maize SHR protein is hypermobile, moving at least eight cell layers into the cortex [54]. These findings suggest that the movement of SHR proteins exhibits diversity when performing functions. The SHR–SCR complex promotes the expression of *WOX5*, which plays a pivotal role in maintaining QC identity and preventing the differentiation of surrounding stem cells [26]. The levels of SHR and SCR early in the cell cycle determine the orientation of the division plane, resulting in either formative or proliferative cell division [55].

SEUSS (SEU) proteins, homologous to the animal LIM-domain binding (LDB) proteins, regulate *WOX5* expression and QC specification. SEU binds to the *WOX5* promoter depending on the transcription factor SCR, and then, SEU physically recruits the SET domain methyltransferase SDG4 to the *WOX5* promoter, thus promoting *WOX5* expression (Figure 1B and Figure 2) [56]. PLT and SCR genetically and physically interact with the plant-specific teosinte-branched cycloidea PCNA (TCP) transcription factors to specify the SCN identity by the modulation of *WOX5* expression (Figure 1B and Figure 2) [43]. The transcription factor NAC1 has been identified as a critical regulator in the asymmetric cell division in root development. Loss of function of *NAC1* results in increased periclinal cell division in the root endodermis. The cell cycle regulator CYCLIND6;1 (CYCD6;1) is a positive regulator in periclinal cell division. NAC1 directly represses the expression of *CYCD6;1* by recruiting the corepressor TOPLESS (TPL) to maintain proper periclinal cell division. NAC1 physically interacts with SCR to restrict excessive periclinal cell divisions in the endodermis during root middle cortex formation. SHR is able to activate *CYCD6;1* expression in the *nac1* mutant. NAC1 and SHR antagonize each other to regulate the expression of *CYCD6;1* [57].

## 3. H_2_O_2_ Homeostasis in RAM Activity Maintenance

The production of H_2_O_2_ depends on superoxide dismutases, plasma membrane NADPH oxidases, peroxisomal oxidases, and apoplastic oxidases, whereas the scavenging of H_2_O_2_ relies on catalase, peroxiredoxin, glutathione peroxidase-like enzymes, and ascorbate peroxidase [21]. Among the factors that regulate H_2_O_2_ homeostasis, NADPH oxidases, known as respiratory burst oxidase homologs (RBOHs), play a major role in H_2_O_2_ production in plants. RBOHs are enzymes located in the plasma membrane, utilizing electrons from cytosolic NADPH to reduce oxygen to O_2_^•−^ in the apoplast. Subsequently, O_2_^•−^ is either spontaneously or enzymatically converted into H_2_O_2_. In Arabidopsis, there are ten RBOHs, each contributing to a diverse array of physiological processes [58]. Increasing salicylic acid (SA) promotes H_2_O_2_ production by enhancing the transcript levels of *RBOHD* and *RBOHF* and affecting QC division in the RAM [24]. Another study also found that SA promotes H_2_O_2_ generation by repressing *CAT2* and *CAT3* expression [59]. Brassinosteroid (BR)-activated transcription factors BRASSINAZOLE RESISTANT1 (BZR1) elevates H_2_O_2_ levels by directly binding to the promoters of *RBOHD* and *RBOHF* [60]. Abscisic acid (ABA) promotes H_2_O_2_ production by the modulation of mitochondria [61,62]. Auxin can induce H_2_O_2_ generation to inhibit root growth and promote lateral root formation [63,64]. Glucose induces H_2_O_2_ accumulation to regulate RAM activity by the modulation of autophagy [65]. Some stresses, such as potassium deprivation and salt stress, can also cause H_2_O_2_ generation in roots [66,67,68]. H_2_O_2_ homeostasis in the RAM is affected by phytohormones and various environmental stresses.

The mutation of *AtRBOHC*, also known as *root hair defective 2* (*rhd2*), results in lower levels of H_2_O_2_ in roots. The primary roots of the *rhd2* mutants become shorter and thinner than those of the wild type [23]. Treatment with diphenylene iodonium (DPI), a specific inhibitor of NADPH oxidase that are used to repress H_2_O_2_ production, can also cause shorter roots [23]. The plastid-localized glutathione reductase2 (GR2) can catalyze the reduction of glutathione disulfide (GSSG) into reduced glutathione (GSH). Loss of function of *GR2* disrupts the redox status of roots, resulting in the strong inhibition of root growth and severe defects in the RAM [69]. Treatment with the reductive reagent GSH or the oxidative reagent H_2_O_2_ has been shown to repress CSC differentiation, suggesting that both highly reductive and oxidative environments inhibit CSC identity [70]. The results suggest that the regulation of H_2_O_2_ homeostasis to maintain appropriate redox status in root tips is critical for RAM activity maintenance.

**Figure 3:**
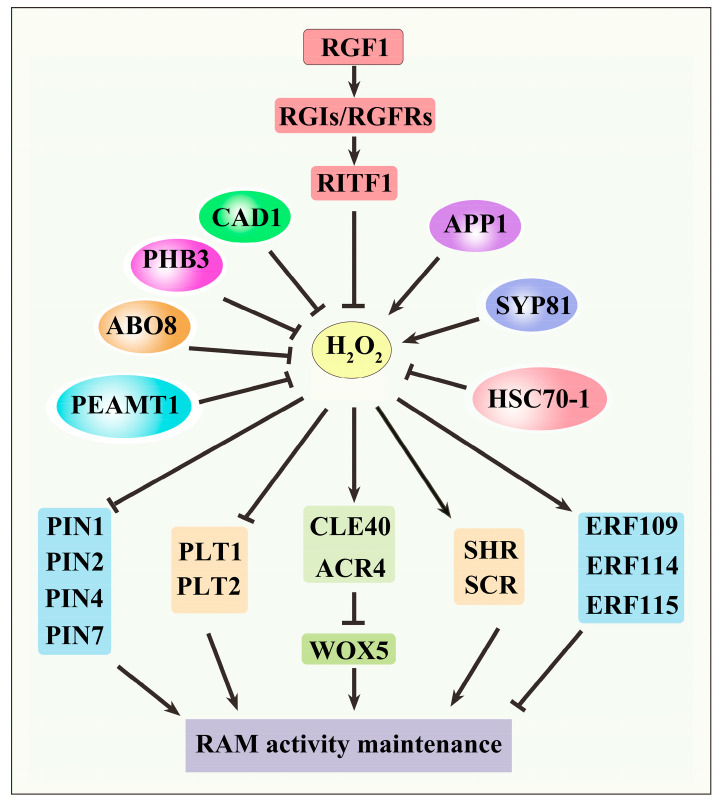
H_2_O_2_ signaling in the regulation of root apical meristem activity. Hydrogen peroxide (H_2_O_2_) plays a crucial role in the regulation of root apical meristem (RAM) activity. Many factors are involved in the regulation of H_2_O_2_ homeostasis. PEAMT1, ABO8, PHB3, CAD1, RITF1, and HSC70-1 contribute to the repression of H_2_O_2_ production, whereas APP1 and SYB81 promote H_2_O_2_ accumulation in the RAM. The factors that induce the alteration of H_2_O_2_ levels regulate RAM activity maintenance via the modulation of PINs, PLTs, SHR, SCR, CLE40, ACR4, and ERFs. H_2_O_2_ homeostasis is important for RAM activity maintenance.

WOX5 is a key player in the determination of QC specification and CSC identity [26]. The receptor-like kinases ACR4/CLV1 negatively regulate CSC identity by repressing *WOX5* expression [30,31]. The mutation of *ACR4* increases the CSC layers, suggesting that ACR4 promotes CSC differentiation [70]. Altering the redox status of RAM using H_2_O_2_ or GSH causes CSC differentiation. The stability of ACR4 is regulated by endocytosis. The tumor necrosis factor receptor (TNFR) is the extracellular domain of ACR4, contributing to the maintenance of the stability of ACR4. ACR4 without the TNFR domain is directly localized to endosomes for degradation, implying that the TNFR domain represses the endocytosis of ACR4. The cysteine residues in the TNFR domain are major determinants of the endocytic degradation of ACR4. The redox status of cysteine in the TNFR domain affects the localization and function of ACR4. The cysteine residues in the TNFR domain play a positive role in ACR4 endocytosis. When treated with GSH or H_2_O_2_, ACR4 endocytosis is detected in the distal stem cell (DSC) of *ProACR4:ACR4-GFP* but not in those of *ProACR4:ACR4* (harboring TNFR12C-A mutations, i.e., all 12 cysteine residues were mutated to Ala)*-GFP*. The multiple cysteine residue mutations in the TNFR of ACR4 block endocytosis. The findings indicate that the cysteine residues in the TNFR domain play a positive role in the redox-mediated internalization and turnover of membrane-localized ACR4. The redox status of the TNFR domain is critical for the localization and function of ACR4. GSH or H_2_O_2_ represses ACR4 function by affecting ACR4 endocytosis via the modulation of the redox status of cysteine in the TNFR domain [70]. CLE40 is the ligand of the ACR4–CLV1 complex and functions as a negative signal to regulate CSC differentiation [30,31]. Altering the redox status using H_2_O_2_ or GSH, the CSC cell layer does not change in the *cle40* mutant. Moreover, the CSC cell layers in the *cle40acr4* double mutant do not change in response to treatment with H_2_O_2_ or GSH, suggesting that both CLE40 and ACR4 participate in the redox regulation of CSC differentiation (Figure 3) [30].

## 4. H_2_O_2_ Signaling in Auxin-Mediated RAM Activity Maintenance

There is a close connection between H_2_O_2_ and auxin in the regulation of root development, including primary root growth, root hair growth, and lateral root formation [23,71,72]. During RAM development, auxin distribution is altered by H_2_O_2_ [23,72]. Auxin gradients in the RAM play a central role in determining QC specification and stem cell identity [37]. The auxin transporters, i.e., PINs contribute to the formation of auxin gradients and auxin maximum in the root tips [37,38]. In the root stele, the transporters, including PIN1, PIN3, PIN4, and PIN7, are responsible for the flux of auxin to the SCN region. PIN1 plays the main role in auxin distribution. In the columella cells, PIN3, PIN4, and PIN7 transport the auxin toward the lateral root cap [4,38]. After treatment with H_2_O_2_, the transcript levels of the auxin transport genes, including *PIN1*, *PIN2*, *PIN7*, and *AUX1*, are repressed [72].

In chloroplasts, superoxide (O_2_^•−^) is predominantly generated through photosystems I and II (PS I and PS II). Meanwhile, in the mitochondria, O_2_^•−^ primarily originates from the respiratory complexes I, II, and III of the electron transport chain (ETC). The O_2_^•−^ produced in both chloroplasts and mitochondria can undergo dismutation, leading to the conversion of O_2_^•−^ to H_2_O_2_. Superoxide dismutase (SOD) plays a crucial role in facilitating this conversion, catalyzing the dismutation of O_2_^•−^ into H_2_O_2_ and O_2_ [49,73,74]. SOD is a kind of metalloenzyme, which can be divided into four types: CuSOD, ZnSOD, FeSOD, and MnSOD. CuSOD, ZnSOD, and FeSOD exist in chloroplasts, and MnSOD is distributed in the mitochondria [21]. Chloroplastic, thylakoid-attached Cu/ZnSOD (chl-Cu/ZnSOD) is encoded by a single gene in Arabidopsis (*At2g28190*). A knock-down mutant of the *At2g28190* gene (*KD-SOD*) contains higher levels of H_2_O_2_ and shows shorter roots with more lateral roots than the wild type [23,75]. Because there are no chloroplasts in roots, it is speculated that the Cu/ZnSOD function may be present in the root plastids. The levels of PIN1, PIN2, and PIN4 proteins are reduced in the *KD-SOD* seedlings. The polarity of PIN1 is impaired by H_2_O_2_ in the division zone of the RAM [23]. These findings suggest that H_2_O_2_ regulates the establishment of the auxin gradient in the RAM by the modulation of PIN protein polarity and abundance (Figure 3) [23].

Phosphoethanolamine N-methyltransferase 1 (PEAMT1) catalyzes phosphocholine biosynthesis in Arabidopsis. The mutation of *PEAMT1* results in the accumulation of H_2_O_2_, leading to RAM consumption and impaired activity of the SCN. The abundance of the auxin transporters, i.e., PIN proteins, including PIN1, PIN2, PIN3, and PIN7, are reduced in the *peamt1* mutant. Moreover, the loss of function of *PEAMT1* impairs PIN2 polar distribution in the root tip. The suppression of ROS over-accumulation partially prevents RAM differentiation in the *peamt1* mutant. PEAMT1 regulates RAM activity by H_2_O_2_-mediated auxin distribution via the modulation of PIN proteins (Figure 3) [76]. The Arabidopsis mitochondrial-localized heat shock protein 70-1 (HSC70-1) regulates polar auxin transport by the modulation of ROS homeostasis in roots. The *HSC70-1* knockout causes severe growth inhibition and an increase in mitochondrial ROS levels [77]. The mutation of *HSC70-1* impairs auxin response and decreases the size and activity of the RAM. The abundance of the auxin transporter carriers, including PIN1, PIN2, PIN3, and PIN7, is reduced in the *hsc70-1* mutant. Treatment with H_2_O_2_ in the wild type has been shown to decrease the transcript levels of the PINs, while treatment with *GSH* in the *hsp70-1* mutant increases the expression levels of the PINs (Figure 3). These results suggest that HSC70-1-mediated ROS homeostasis regulates auxin distribution by affecting the expression of *PINs* [78].

Root survival under flooding-induced hypoxic stress depends on the maintenance of QC activity within the RAM, which is controlled by an auxin maximum. Hypoxic stress causes the disruption of auxin transport and auxin maximum formation and shifts the redox state of the QC towards a more reduced environment, leading to the activation of QC, degradation of the meristem, and root abortion. The maize phytoglobin gene *ZmPgb1.1* minimizes the damaging effects under hypoxic stress. *ZmPgb1.1* contributes to sustaining the PIN-mediated auxin maximum and an oxidized environment in the QC. The findings suggest that maintaining the redox state of QC is critical for root development under stresses [79].

## 5. H_2_O_2_ Signaling in PLT-Mediated RAM Activity Maintenance

Root meristem growth factors (RGFs), a family of functionally redundant homologous peptide hormones, have been documented as crucial regulators of RAM development. RGF1 is a peptide that regulates RAM size, and treatment with RGF1 in the Arabidopsis roots can increase RAM size, while the *rgf1/2/3* triple mutant has a smaller RAM size [80]. Using genetic approaches, the researchers identified a clade of leucine-rich repeat receptor-like kinases, designated as RGF1 INSENSITIVE 1 (RGI1) to RGI5, also named RGF1 RECEPTORS (RGFRs), acting as the receptors of RGF1. The *rgi1rgi2rgi3rgi4rgi5* quintuple mutant displays a small RAM and is completely insensitive to RGF1. The expression of *PLT1* and *PLT2* is almost undetectable in the quintuple mutant. RGF1 and its receptors RGIs regulate RAM activity by modulating the expression of *PLT1* and *PLT2* (Figure 3) [81]. To find the proteins connecting RGF1-RGIs, the mitogen-activated protein (MAP) kinases kinase 4 (MKK4) and kinase 5 (MKK5), along with their downstream targets MPK3 and MPK6, have been identified as the essential RGI-dependent regulators of RAM development. The MKK4/MKK5-MPK3/MPK6 module functions downstream of YDA, a MAPKKK. RGF1-RGI1 regulates the expression of *PLT1* and *PLT2* via a YDA-MKK4/MKK5-MPK3/MPK6 signaling cascade [82,83]. RGF1 can induce the expression of *RGF1 INDUCIBLE TRANSCRIPTION FACTOR1* (*RITF1*), which is predominantly active in the meristematic zone of the root tip. RITF1 plays a pivotal role in mediating the signaling cascade initiated by RGF1. The overexpression of *RITF1* reduces H_2_O_2_ levels in roots and increases the O_2_^•−^ signal of the root meristematic zone, which enhances the stability of the PLT2 protein to increase root meristem size (Figure 3) [84].

*SYNTAXIN OF PLANTS81* (*AtSYP81*) is a syntaxin gene identified in *Arabidopsis thaliana*. Staining with DAB and NBT indicated a reduction in H_2_O_2_ and O_2_^•−^ levels in the *atsyp81* mutants. The application of H_2_O_2_ or O_2_^•−^ suppresses QC cell division and prevents the differentiation of CSCs in the primary root SCN of *atsyp81-1* seedlings. An analysis of RNA-Seq data and qRT-PCR revealed that a subset of genes encoding class III peroxidases (Prxs) are upregulated in *atsyp81-1* seedlings. Subsequent investigations elucidated PLT1/PLT2 as downstream elements involved in the regulation of ROS homeostasis and the activity of the RAM mediated by AtSYP81 [25]. This comprehensive analysis provides valuable insights into the intricate molecular mechanisms governed by AtSYP81 in plant growth and development (Figure 3). Prohibitins (PHBs) are recognized as having a potential tumor suppressor role with anti-proliferative activity. It has been shown that the *phb3* mutant displays a short-root phenotype [85]. Loss of function of PHB3 results in enhanced root DSC differentiation and increased mitotic activity in QC. *PHB* overexpression lines display additional cell layers in the DSC. The mutation of *PHB3* causes an accumulation of ROS, including H_2_O_2_ and O_2_^•−^. Three ROS-responsive ethylene response factor (ERF) genes, namely, *ERF109*, *ERF114*, and *ERF115*, are upregulated in the *phb3* mutant. The mutation of *PHB3* also represses the transcription of *PLT1* and *PLT2* by increasing ROS levels. Both *ERF* genes and *PLT* genes are key ROS-responsive factors in the mediation of RAM activity (Figure 3) [86].

SA and ABA play a critical role in QC maintenance. The SA overaccumulation mutant constitutively activated cell death 1 (*cad1*) exhibits increased cell division in QC. The application of exogenous SA also promotes QC cell division. SA promotes H_2_O_2_ and O_2_^•−^ production, which is important for QC cell division. SA regulates QC maintenance largely by the ROS-mediated repression of the expression of *WOX5*, *PLT1*, and *PLT2* (Figure 3) [24]. An ABA overly sensitive mutant, *abo8-1*, which is defective in a pentatricopeptide repeat (PPR) protein responsible for the splicing of NAD4 intron 3 in the mitochondrial complex I, accumulates more ROS in root tips. The mutation of *ABO8* reduces RAM activity, which can be recovered by the reducing agent GSH. The expression of *PLT1* and *PLT2* is significantly reduced in the *abo8-1* mutant. ABO8 regulates RAM activity by the modulation of *PLT* expression via ROS homeostasis (Figure 3) [61].

## 6. H_2_O_2_ Signaling in SHR–SCR Module-Mediated RAM Activity Maintenance

The *Arabidopsis thaliana* P-loop NTPase APP1 exerts regulatory control over RAM identity via the ROS-mediated SHR–SCR pathway. The *app1* mutant exhibits aberrant cell divisions in the QC and impairs DSC identity. The mutation of *APP1* results in reduced levels of H_2_O_2_ and O_2_^•−^ in the root tip, suppressing the expressions of *SCR* and *SHR* and consequently increasing the rate of QC cell division and root DSC differentiation. Conversely, elevated ROS levels in the *APP1*-overexpression line promote QC cell division and root DSC differentiation. The expression of *PLT1* and *PLT2* is downregulated in the presence of heightened ROS levels, suggesting that altered ROS levels may modulate RAM activity through distinct pathways (Figure 3) [87].

SHR and SCR are important for the maintenance of a healthy redox status in roots. The mutation of *SHR* or *SCR* results in ROS accumulation, probably due to decreased expression of *peroxidase gene 3* (*PER3*) [88]. SHR is capable of establishing an optimal microenvironment for periclinal division in cortex endodermal initial cells by maintaining ROS homeostasis in Arabidopsis roots. SHR achieves this by significantly increasing ROS levels, primarily through the promotion of *RESPIRATORY BURST OXIDASE HOMOLOG* (*RBOH*) expression. Scavenging H_2_O_2_ has been demonstrated to markedly impair SHR’s ability to induce periclinal division. Interestingly, H_2_O_2_, rather than O_2_^•−^, plays a crucial role in SHR-mediated periclinal division [59]. Recent investigations have revealed an interaction between SHR and BRASSINAZOLE RESISTANT1 (BZR1), a crucial transcription factor in the BR signaling pathway. Both BZR1 and SHR directly bind to the promoters of *RBOHD* and *RBOHF*, thereby promoting their expression and subsequently increasing H_2_O_2_ accumulation. This elevated H_2_O_2_ level, in conjunction with the presence of BZR1, has the capacity to induce periclinal division. These findings suggest a high level of coordination between BR and H_2_O_2_ signals in finely regulating the periclinal division of cortex endodermal initials through the BZR1–SHR module [60].

## 7. Conclusions

H_2_O_2_ homeostasis is critical for the maintenance of RAM activity, and increased or decreased levels of H_2_O_2_ have important effects on auxin biosynthesis, transport, and signaling. However, the direct regulation of H_2_O_2_ on auxin signaling is not well understood. H_2_O_2_ can directly regulate the function of its target proteins by oxidative post-translational modifications. H_2_O_2_ reacts with the sulfur atoms of systeine and methionine residues to form S-sulfenylated, S-nitrosated, or persulfidated residues [89]. The identification of new direct targets of H_2_O_2_ needs to be investigated further. Additionally, single-cell RNA sequencing has been used to study root development in Arabidopsis, maize, and rice [54,90,91,92,93]. The advancement of new technologies provides new insights into understanding the function of H_2_O_2_ in the maintenance of the RAM.

RAM maintenance is very important for root development and stress adaptation. H_2_O_2_ acts as a critical signal to help RAM maintenance. The homeostasis and signaling of H_2_O_2_ are crucial for stem cell specification and formative cell division. Many key players, such as *PLTs*, *SCR*, *SHR*, and *PINs*, are regulated by H_2_O_2_ in RAM development; however, the fine-tuned regulatory mechanisms of H_2_O_2_ controlling the core factors need to be studied in the future. It will be interesting to elucidate the redox regulation of proteins in H_2_O_2_-mediated RAM development.
